# Time Course Characterization of Protective Immune Responses Following BCG Vaccination in BALB/c Mice

**DOI:** 10.3390/pathogens15040392

**Published:** 2026-04-06

**Authors:** Hee Ho Kim, Kwangwook Kim, Min Jung Kim, Ye Jin Yang, Eun Bee Choi, Ji Woong Heo, Seo Young Moon, Heeji Lim, Yookyoung Lee, In-Ohk Ouh, Kwang Il Park

**Affiliations:** 1 College of Veterinary Medicine, Gyeongsang National University, Gazwa, Jinju 52828, Republic of Korea; hiho2323@gnu.ac.kr (H.H.K.); minjung0102@gnu.ac.kr (M.J.K.); yang93810@gnu.ac.kr (Y.J.Y.); hujiw7806@gnu.ac.kr (J.W.H.); 2 Division of Vaccine Development Coordination, Center for Vaccine Research National Institute of Infectious Diseases, Korea National Institute of Health, Korea Disease Control and Prevention Agency, Cheongju-si 28159, Republic of Korea; kwkim83@korea.kr (K.K.); dmsql2274@korea.kr (E.B.C.); msy1477@korea.kr (S.Y.M.); dalgi0519@korea.kr (H.L.); leeykyoung@korea.kr (Y.L.)

**Keywords:** Bacillus Calmette–Guérin (BCG), tuberculosis, immunization period, immune response

## Abstract

Background/Objectives: Tuberculosis (TB) remains a major global health challenge, requiring standardized animal models to evaluate vaccine-induced immune responses. This study characterized time-dependent immune responses following Bacillus Calmette–Guérin (BCG) vaccination in BALB/c mice. Methods: BALB/c mice were vaccinated with BCG, and the immune responses and protective efficacy were evaluated at 4, 6, and 8 weeks post-immunization. The cytokine expression in serum, lung, and spleen tissues was analyzed using ELISA, quantitative PCR, and immunohistochemistry. Protective efficacy was assessed via colony-forming unit (CFU) enumeration and the immunohistochemical detection of Mycobacterium TB after aerosol challenge. Results: The BCG vaccination induced time-dependent and tissue-specific cytokine responses. Pulmonary IL-1β and splenic IFN-γ levels were significantly increased four weeks post-vaccination. At 8 weeks, serum IL-2, pulmonary IL-2, and TNF-α were significantly increased, whereas no significant changes in cytokines were observed at 6 weeks. After the challenge, BCG-vaccinated mice exhibited reduced bacterial burdens compared with controls, but the differences among the 4-, 6-, and 8-week groups were modest. Conclusions: Immune responses became detectable starting four weeks after BCG vaccination, with temporal differences observed in cytokine expression. Week 8 may serve as a reference point for monitoring cytokine dynamics rather than as an optimal time for protection.

## 1. Introduction

Tuberculosis (TB) is an infectious disease that is primarily transmitted through the air and caused by the *Mycobacterium tuberculosis* (*M. tuberculosis*) complex [1]. Granuloma formation is a major pathological feature in the lungs and helps suppress the spread of the disease [2,3,4].

To date, the Bacillus Calmette–Guérin (BCG) vaccine remains the only approved vaccine for TB and is administered to children [5,6]. However, the persistence of individual immune variability, latent tuberculosis infections (LTBIs) and multidrug-resistant tuberculosis (MDR-TB) continues to impede efforts to eradicate the disease [7,8]. Consequently, TB remains a significant public health concern [1,9] and new vaccine candidates are being actively investigated [10].

The BALB/c mouse is a commonly used laboratory model due to its ease of handling and high reproducibility [11,12,13,14]; it is favored for the evaluation of new TB vaccine candidates. However, the schedule for immunological assessments has not been consistent across studies [15,16,17,18,19]. Such heterogeneity in experimental timelines complicates direct comparisons of immune readouts and protective efficacy. Consequently, these variations hinder consistent evaluations and pose operational challenges for the development of new TB vaccines.

In this context, with the present study we aimed to investigate the immune responses induced by BCG vaccination in BALB/c mice. The Th1 immune response is a major contributor to host protection against TB; however, Th2-associated responses are also involved in modulating inflammation and shaping the overall immune environment during infection. Consequently, recent studies indicate that standard infant and murine BCG doses elicit a mixed Th1/Th2 profile [8]. There is emerging evidence that TB vaccine candidates designed to enhance humoral immunity can confer protection, further highlighting the importance of maintaining an appropriate Th1/Th2 balance [16,17,18,19].

Nevertheless, the criteria for evaluating candidates’ efficacy remain insufficient, leading to inconsistencies that hinder the reliable assessment of their protective functions. Accordingly, we analyzed immune responses at multiple post-vaccination time points to elucidate the temporal dynamics of protective immunity against TB. These results characterize the onset of protective responses across the serum, spleen, and lungs and provide a strengthened framework to assess vaccine candidates.

## 2. Materials and Methods

### 2.1. Ethics and Animal Care

Five-week-old female BALB/c mice were acquired from SAMTAKO (Osan, Gyeonggi, Republic of Korea). Mice were housed under a 12 h light/dark cycle at a temperature of 22 ± 2 °C and a relative humidity of 71.5 ± 5%. All mice were utilized after a week’s adaptation period and were monitored daily by trained technicians, with increased frequency if adverse reactions were noted. No adverse effects or >20% body weight loss occurred in any animal throughout the study. All procedures were conducted in accordance with ARRIVE guidelines. This study was approved by the Institutional Animal Care and Use Committee (IACUC) of the Korea Disease Control and Prevention Agency (KDCA-IACUC-21-034).

### 2.2. BCG Vaccination, M. tuberculosis Infection, and Sample Collection

**Figure 1:**
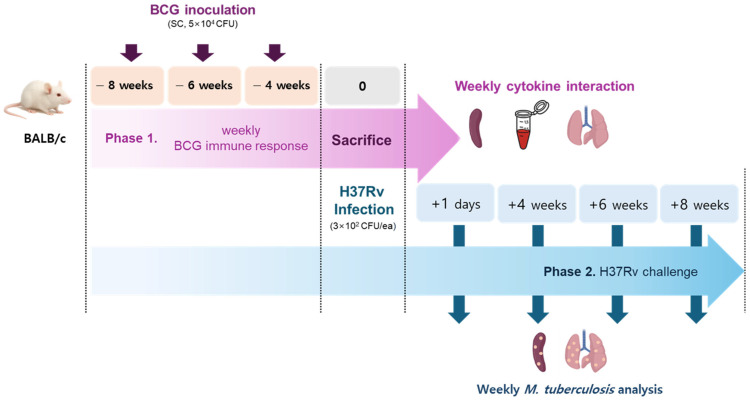
Schedule of BCG vaccination and *M. tuberculosis* challenge.

This study consisted of two phases: weekly assessments of BCG immune responses and *M. tuberculosis* inhibition. In line with the established schedule (Figure 1), five mice per group were vaccinated via subcutaneous inoculation with BCG Pasteur 1173P2 (5 × 10^4^ CFU), while phosphate-buffered saline (PBS) was administered to the control group. At 8 weeks post-initiation, five mice from each vaccinated group (−8 weeks, −6 weeks, and −4 weeks) were sacrificed simultaneously. Serum and tissue samples were collected to assess week-dependent cytokine interactions between organs. On the same day, five mice per group (+1 day, +4, +6, and +8 weeks) were infected with *M. tuberculosis* H37Rv via aerosol and sacrificed according to the schedule. Spleen and lung tissues were collected for assessment. All groups received the same aerosol challenge dose of *M. tuberculosis* H37Rv at a common infection time point. Vaccination schedules were defined relative to this fixed challenge day, and post-infection analyses were conducted at identical intervals (+1 day, +4, +6, and +8 weeks) across groups to enable direct comparison.

### 2.3. ELISA Analysis

Serum levels of IL-2, TNF-α, and IFN-γ were measured to assess the immune response triggered by BCG vaccination. Cytokine concentrations were quantified as absolute levels (pg/mL) using a commercially available ELISA kit (R&D Systems, Minneapolis, MN, USA; DY402-05 for IL-2; DY410-05 for TNF-α; and DY485-05 for IFN-γ) according to the manufacturer’s instructions.

### 2.4. qPCR Analysis

Total RNA was extracted from spleen and lung tissue samples of BCG-vaccinated mice, following the schedule shown in Figure 1, using an RNase mini kit (Thermo Fisher Scientific, Waltham, MA, USA, 12183020) and TRIzol™ (Thermo Fisher Scientific, Waltham, MA, USA, 10296028). Genomic DNA was removed using DNase I, and the isolated total RNA was analyzed by qPCR with the Power SYBR™ Green RNA-to-CT™ 1-Step Kit (Thermo Fisher Scientific, Waltham, MA, USA, 4389986) on an Applied Biosystems 7500 Fast Real-Time PCR System (Thermo Fisher Scientific, Waltham, MA, USA, 4351104). Relative gene expression levels were calculated using the 2^−ΔΔCt^ method. Expression of target genes was normalized against the housekeeping genes 18S rRNA, β-actin, and GAPDH, and results were expressed relative to the control group.

### 2.5. Bacterial Culture

For the challenge experiments, *M. tuberculosis* H37Rv was used as the reference strain. The *Mycobacterium bovis* BCG Pasteur 1173P2 strain was used as the TB vaccine. Each strain was cultured on Middlebrook 7H10 agar medium at 37 °C for a period of two to three weeks. Subsequently, a seed culture was established by inoculating 10 mL of 7H9 broth medium supplemented with 10% oleic acid-albumin-dextrose-catalase (OADC) and 0.05% Tween 80, which was then incubated for one week. The culture was subsequently transferred to 400 mL of fresh 7H9 broth medium and incubated for an additional week. Once the optical density (OD600) reached between 0.6 and 0.8, each mycobacterial culture was harvested and filtered through a 10 μm cell strainer to isolate single bacterial cells. The isolated cells were completely resuspended in 8 mL of 7H9 broth, followed by the addition of 20% glycerol. The bacterial suspensions were aliquoted into 1 mL stock tubes and stored at −70 °C until use.

### 2.6. M. tuberculosis H37Rv Infection

To establish the mouse model of TB infection, mice were aerosolized with the *M. tuberculosis* H37Rv strain using a Glas-Col^®^ aerosol exposure chamber system (Glas-Col, Terre Haute, IN, USA). A 3 mL suspension of the bacterial strain, diluted to 3 × 10^2^ colony-forming units (CFUs)/mL, was dispensed into a glass vial a nebulizer, and aerosol infection was performed under the conditions specified in Table 1.

### 2.7. CFU Enumeration

As illustrated in Figure 1, mice infected with *M. tuberculosis* H37Rv were euthanized at various time points post-infection: 1 day, 4 weeks, 6 weeks, and 8 weeks. Lung and spleen tissues were then harvested from each group. The harvested tissues were homogenized and serially diluted (10^2^–10^5^-fold dilutions in PBS). Aliquots of these dilutions were subsequently inoculated into 7H9 broth medium containing 10% OADC and 0.05% Tween 80. Following incubation at 37 °C for 2–3 weeks, the number of colonies was counted to determine the CFUs. Homogenization was carried out using a Gentle MACS^TM^ dissociator in accordance with the manufacturer’s instructions.

### 2.8. H&E Staining

Lung and spleen tissues, sectioned to thicknesses of less than 3 mm, were fixed in 10% formalin for 24 to 48 h. The tissues, embedded in paraffin blocks, were then sectioned into 5–8 μm slices. After deparaffinization with xylene and ethanol, the tissues were stained using hematoxylin and eosin. Histopathological evaluation was performed in a blinded manner by independent investigators who were unaware of the group allocations. Inflammation severity was assessed using a semi-quantitative scoring system (Table 2) adapted from a previously published study [20].

### 2.9. IHC Staining

Lung and spleen tissues were fixed, embedded in paraffin, and then sectioned at a thickness of 3 μm onto glass slides. Paraffin was subsequently removed using xylene, followed by rehydration through successive washes with alcohol and distilled water. To retrieve the antigens, slides were immersed in a 10 mM sodium citrate buffer (pH 6.0) and then heated in a microwave. After cooling sufficiently at room temperature, the slides were washed with PBS. Endogenous peroxidase activity was inhibited using 3% hydrogen peroxide dissolved in methanol. This was followed by a 1 h blocking with 5% bovine serum albumin. Following an overnight incubation with the primary antibody at 4 °C, the slides were washed with PBS and then incubated with the secondary antibody for 2 h at room temperature. The primary antibodies were anti-IL-2 (Biomatik, Cambridge, ON, Canada), anti-TNF-α (Abcam, Cambridge, UK), anti-IFN-γ (MyBioSource, San Diego, CA, USA), and anti-*M. tuberculosis* (Abcam, Cambridge, UK). The secondary antibodies used were either biotinylated goat anti-rabbit IgG or rabbit anti-rat IgG (1:100, Vector Laboratories, Burlingame, CA, USA). Following 2 h of incubation with the avidin-biotinylated enzyme complex at room temperature, the slides were washed with PBS. Color development was achieved by a treatment with diaminobenzidine tetrahydrochloride. For analysis, the slides were counterstained with Harris hematoxylin and were examined under an optical microscope at 200× magnification. The number of nuclei-stained reddish brown were manually counted and quantified using ImageJ (v1.54k, National Institutes of Health, Bethesda, MD, USA).

### 2.10. Statistical Analysis

Statistical analyses were performed using GraphPad Prism (version 8.02; GraphPad Software, San Diego, CA, USA). Data are presented as mean ± standard deviation (SD). Differences among groups were evaluated using one-way analysis of variance (ANOVA) followed by Dunnett’s multiple comparison test. Exact *p*-values are reported where applicable. Given the small sample size, the results should be interpreted with caution as the study was not powered to detect small effect sizes. A *p*-value < 0.05 was considered statistically significant.

## 3. Results

### 3.1. IL-2 Surge Was Observed at 8 Weeks Post Vaccination in Serum

**Figure 2:**
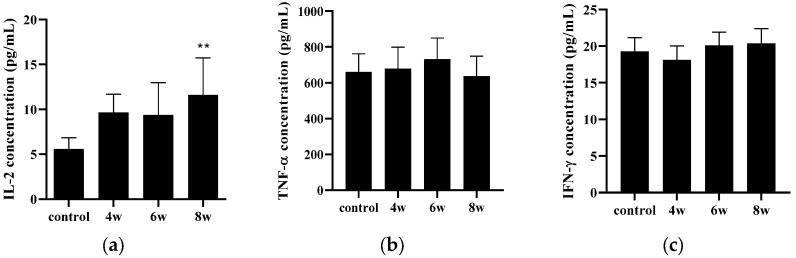
Serum cytokine expression levels following BCG vaccination. IL-2, TNF-α and IFN-γ levels were measured in serum isolated from BCG vaccinated BALB/c mice. Serum samples collected from each mouse group were analyzed by ELISA for (**a**) IL-2 protein levels, (**b**) TNF-α protein levels, and (**c**) IFN-γ protein levels. Data represent the mean ± SD (ANOVA, ** *p* < 0.01). Each group was compared with the control group.

Serum analysis reflects the systemic immune status and plays a key role in mediating immune responses from the spleen to TB infected organs [21,22,23]. Therefore, serum analysis is widely applied for the purpose of evaluating the efficacy of vaccines and therapeutics. Figure 2a shows a significant spike in serum IL-2 levels in BALB/c mice 8 weeks following the BCG vaccination. Statistically significant changes in serum TNF-α and IFN-γ were not identified (Figure 2b,c).

### 3.2. Weekly Cytokine Shifts Elucidated Following BCG Vaccination

**Figure 3:**
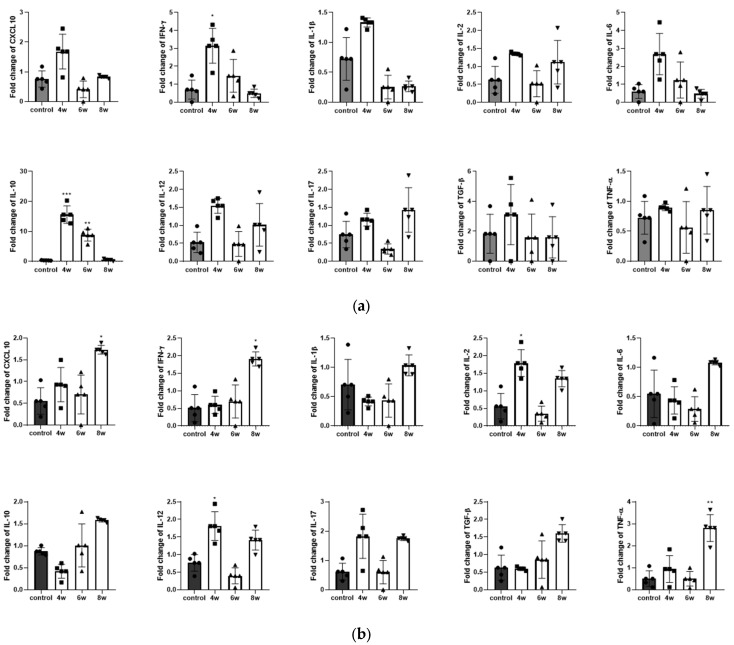
Cytokines and chemokines gene expression in BALB/c lung and spleen following BCG vaccination. (**a**) Expression of the CXCL10, IFN-γ, IL-1β, IL-2, IL-6, IL-10, IL-12, IL-17, TGF-β and TNF-α genes in lung tissues following BCG vaccination. (**b**) Expression of the CXCL10, IFN-γ, IL-1β, IL-2, IL-6, IL-10, IL-12, IL-17, TGF-β and TNF-α genes in spleen tissues following BCG vaccination. The *x*-axis represents the time points at 4–8 weeks following BCG administration, whereas the *y*-axis represents the relative gene expression levels. Black, white, and gray bars represent different experimental groups. Symbols indicate statistical significance. Data represents the mean ± SD of five mice (ANOVA, * *p* < 0.05, ** *p* < 0.01, *** *p* < 0.001).

The lungs are the primary target for TB, which is transmitted via aerosols, and the spleen is a major lymphoid organ involved in immune responses [24]. The BCG vaccination induced distinct week-specific changes in the cytokine gene expression in the lungs and spleen. In the lungs, an elevation of IFN-γ and IL-10 was prominent at 4 weeks after the BCG vaccination (Figure 3b). These cytokines subsequently exhibit a steep decline, with expression levels returning to baseline by week 8 (Figure 3a). Significantly, IL-10 suppression in the lungs was evident following vaccination (Figure 3a). In the spleen, as a first finding, an increase in IL-2 and IL-12 were confirmed 4 weeks after the BCG vaccination (Figure 3b). These critical Th1-inducing cytokines, which are known for their dual functions, exhibited a temporal pattern characterized by a decrease at week 6 and a rebound at week 8 (Figure 3b). In addition, CXCL10, IFN-γ, and TNF-α clearly peaked 8 weeks after the BCG vaccination (Figure 3b).

### 3.3. Tissue-Specific Profiles of IL-2, TNF-α and IFN-γ in the Lung and Spleen for TB Control

**Figure 4:**
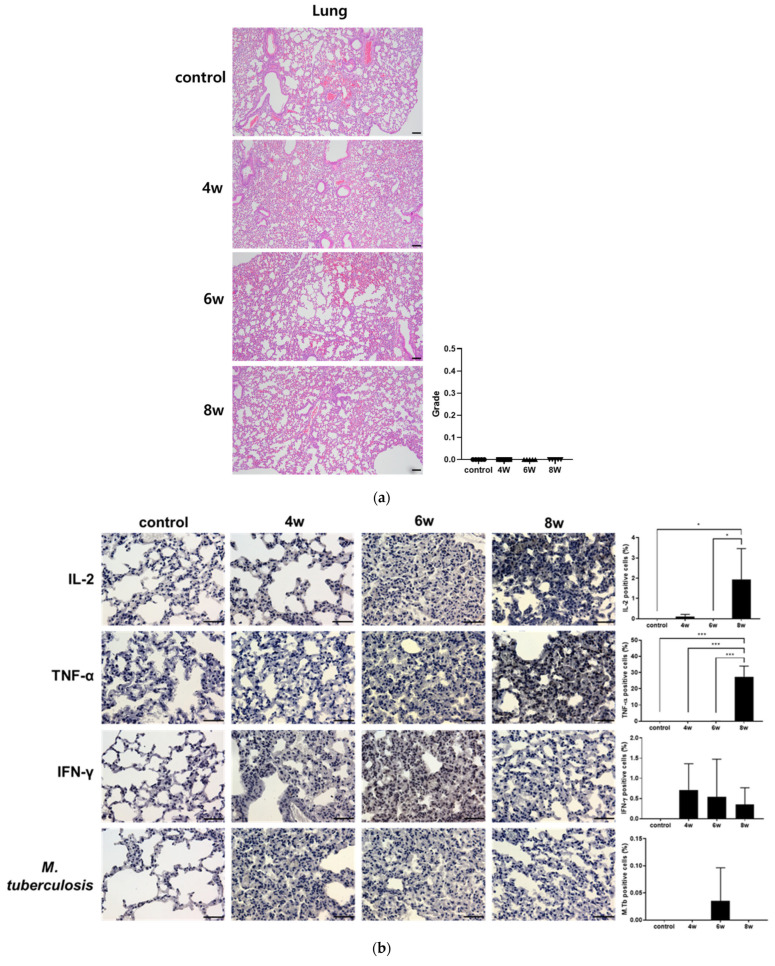

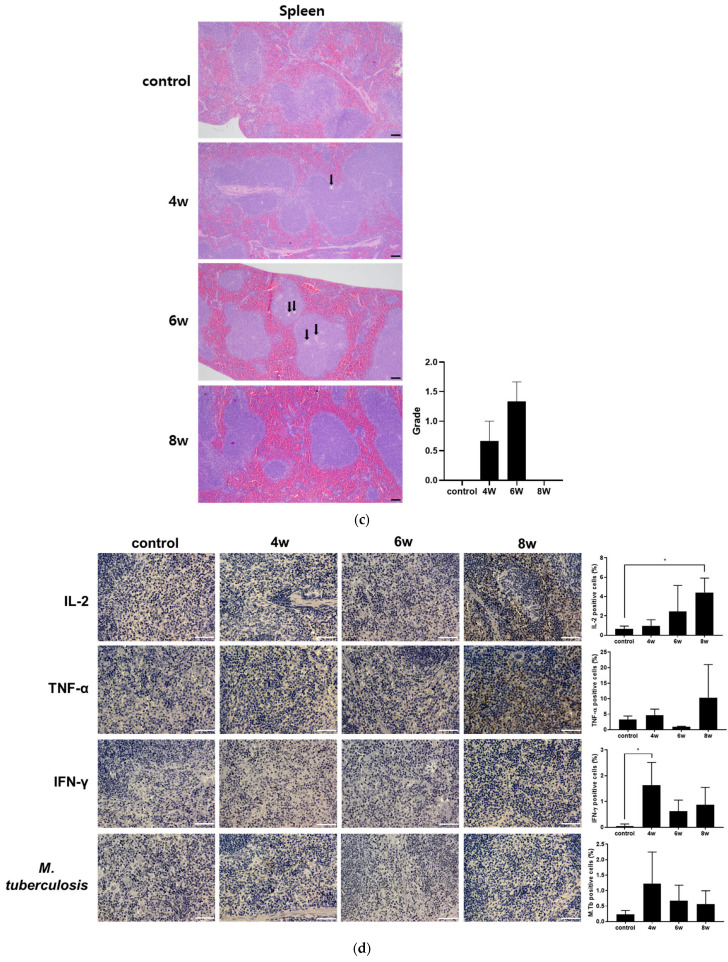
Tissue staining results following BCG vaccination. (**a**) H&E staining of lungs following BCG vaccination. (**b**) IHC of IFN-γ, IL-2, TNF-α and *M. tuberculosis* in BALB/c lung. (**c**) H&E staining of spleen following BCG vaccination. Aggregates of inflammatory cells with macrophage-like morphology are indicated by black arrows. (**d**) IHC of IFN-γ, IL-2, TNF-α and *M. tuberculosis* in BALB/c spleen. Representative microphotographs are shown for each experimental group. Quantification of IHC-positive cells was performed by analyzing multiple randomly selected microscopic fields per tissue section. The number of positive cells for IHC was compared with those in the control and BCG groups. Data represents the mean ± SD of five mice (ANOVA, * *p* <0.05, *** *p* < 0.001), Scale bar = 100 μm.

IL-2, TNF-α, and IFN-γ are understood to be pivotal cytokines elicited by BCG vaccination in BALB/c mice [25]. Over the post-vaccination weeks, the expression levels exhibited striking differential patterns between the lung and spleen samples (Figure 4b,d). In the lungs, an explosive surge of IL-2 and TNF-α was noted 8 weeks post-BCG vaccination. A uniform reduction in the TB bacterial load was observed in the spleen and lungs 8 weeks after vaccination. In the spleen, immunohistochemistry (IHC) results revealed a marked increase in IFN-γ 4 weeks after the BCG vaccination, which subsequently declined. As for IL-2, its expression progressively increased, peaking at week 8. Furthermore, no histopathological lesions were observed in either the lungs or the spleen at week 8 (Figure 4a,c). However, mild macrophage aggregation was induced in the spleen at weeks 4 and 6 (Figure 4c).

### 3.4. BCG Vaccination Effectively Suppressed TB Regardless of Vaccination Period

**Figure 5:**
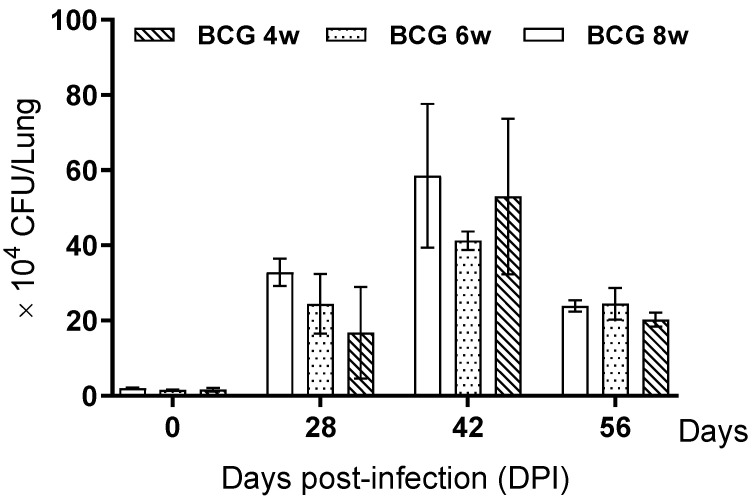
CFU results following *M. tuberculosis* H37Rv challenge in lung. BCG vaccination for eight weeks effectively inhibits the growth of *M. tuberculosis* in the lungs of BALB/c mice. The CFU inhibition effect of BCG vaccine was measured according to the duration of *M. tuberculosis* H37Rv infection. *M. tuberculosis* growth was measured at day 1, four weeks, six weeks and eight weeks in BALB/c lung samples. Day 1 means the basal level of *M. tuberculosis*. Data represents the mean ± SD of five mice.

Lung tissues were collected from TB-infected mice based on a weekly BCG vaccination schedule, as shown (Figure 1); samples were collected at specific time points (day 1, 4 weeks, 6 weeks, and 8 weeks post-challenge). The bacterial burden was quantified via CFU measurement. However, the differences in the bacterial burden across BCG vaccination weeks were found to be marginal (Figure 5). Independent of the BCG vaccination week, the pulmonary bacterial burden demonstrated a consistent rise from day 1 to week 6 post-infection, followed by a plateauing of the bacterial load. An exception was observed in the 6-week BCG vaccination group, which demonstrated the lowest bacterial burden at 6 weeks post-challenge.

### 3.5. BCG Efficacy: Consistency Across 4, 6, and 8 Weeks of Immunization

**Figure 6:**
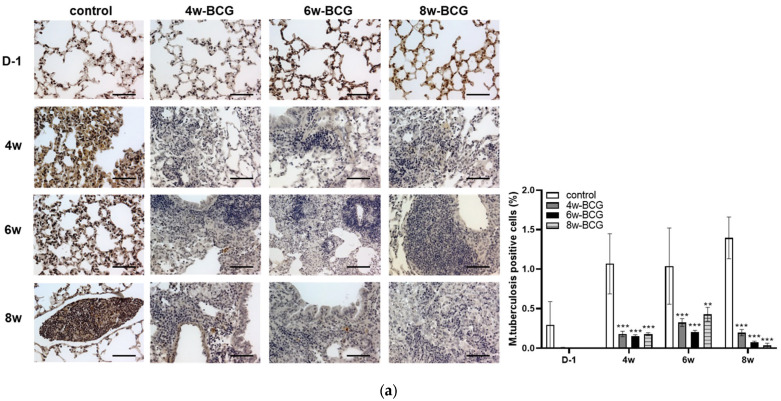

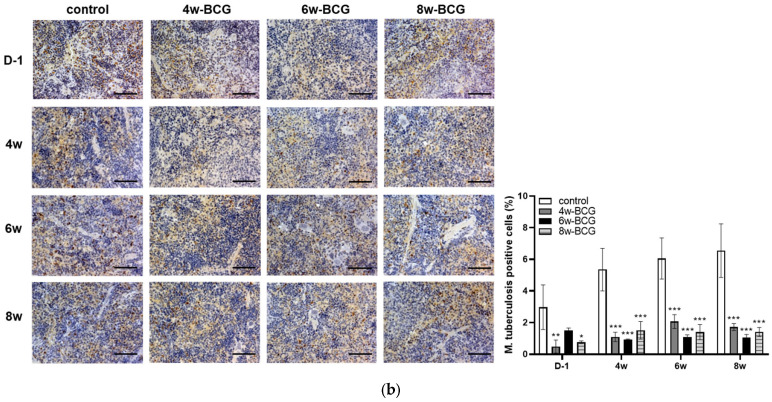
IHC results following *M. tuberculosis* H37Rv infection. (**a**) IHC of *M. tuberculosis* in BALB/c lung. (**b**) IHC of *M. tuberculosis* in BALB/c spleen. All experimental groups were vaccinated with BCG, and mice were subsequently challenged with *M. tuberculosis* H37Rv at the indicated time points, except for the control group, which received BCG vaccination but was not infected with *M. tuberculosis*. Representative microphotographs are shown, and quantification of IHC-positive cells was performed by analyzing multiple randomly selected fields per tissue section. The number of positive cells for IHC was compared with those in the control and BCG groups. Different bar colors represent experimental groups, and statistical significance is denoted by symbols. Data represents the mean ± SD of five mice (ANOVA, * *p* <0.05, ** *p* <0.01, *** *p* < 0.001), Scale bar = 100 μm.

IHC for *M. tuberculosis* was performed on the spleen and lungs at multiple time points (day 1, 4 weeks, 6 weeks and 8 weeks post-infection) to evaluate the protective efficacy of the BCG vaccine. The vaccination provided a significant protective effect against TB at all measured time points, irrespective of the vaccination week (Figure 6). These results were consistently observed in both the spleen and the lungs.

## 4. Discussion

TB is caused by an intracellular bacterium that infects and persists within immune cells, thereby establishing infection [4]. Through various survival strategies within immune cells, *M. tuberculosis* induces the necrosis of infected cells and the subsequent recruitment of new immune cells, establishing an infection that is notoriously difficult to eradicate completely [26,27]. The major protective immunity against TB is primarily mediated by a Th1 immune response [2,28,29]. Consequently, this response serves as a key indicator for the evaluation of various novel TB vaccine candidates, as well as the BCG vaccine [30]. Specifically, the effective induction of such an immune response is a primary objective for both the established BCG vaccine and novel TB vaccine candidates.

IFN-γ, TNF-α, and IL-2 are critical cytokines that sustain and amplify Th1 immune responses [31,32]. Furthermore, IL-12 and IL-1β play crucial roles in initiating the early Th1 immune response [33,34,35], while the Th17 response also contributes significantly to the initial host defense by activating neutrophils [36,37]. TNF-α and CXCL10 are known to contribute to effective granuloma formation by recruiting activated immune cells to the site of the infection [38,39]. Conversely, IL-10 and TGF-β are the primary cytokines responsible for immunosuppression [40,41,42,43], inhibiting granuloma formation and thereby leading to latent infection if immunosuppression persists [44].

Research has extensively elucidated the functions of Th1 cytokines [45], which are now widely employed for evaluating the efficacy of novel TB vaccines [46]. However, a dearth of research on the precise timing of adaptive immune induction following vaccination has led to inconsistencies in the immunization observation schedules employed in the evaluation of various vaccine candidates to date. This represents one of the factors that impede efficient vaccine evaluations and hinder communication among researchers. Accordingly, this study was conducted with the objective of elucidating the precise timing of adaptive immune induction in various major organs.

This experiment was conducted to standardize the assessment of BCG immunization in BALB/c mice by evaluating immune responses at multiple time points. As shown in Figure 2, Figure 3 and Figure 4, cytokine alterations were observed in a time-dependent and tissue-specific manner following BCG vaccination. A significant increase in pulmonary IL-1β (Figure 3a) and splenic IFN-γ (Figure 4d) was detected at four weeks post-vaccination. At eight weeks post-vaccination, a significant increase in serum IL-2 (Figure 2a) was observed, along with elevated pulmonary IL-2 and TNF-α and increased splenic IL-2 (Figure 4b,d). No significant cytokine changes were detected at six weeks post-vaccination. The histological examination revealed no apparent BCG-induced changes in the lung tissue, whereas macrophage aggregation was observed in the spleen at four and six weeks but not at eight weeks (Figure 4a,c). Following the *M*. *tuberculosis* challenge, CFUs increased from day 1 to six weeks and then decreased by eight weeks post-infection (Figure 5). Although the six-week immunization group showed a relatively lower CFU value at six weeks post-infection, the bacterial burden at eight weeks post-infection was comparable across immunization periods of four weeks or longer. Consistent with these findings, the IHC analysis revealed fewer *M. tuberculosis*-positive cells in lung and spleen tissues of BCG-vaccinated groups compared with the controls (Figure 6).

These findings indicate that BCG-induced immune responses and bacterial control in BALB/c mice occur in a time-dependent manner. Although IFN-γ, a hallmark Th1 cytokine, exhibited an increasing trend following the BCG vaccination, this change did not consistently reach statistical significance. In contrast, significant increases in IL-2 (*p* < 0.05) and TNF-α (*p* < 0.001) were observed at eight weeks post-vaccination. *M. tuberculosis* infection and the associated host immune responses have been reported to influence the production of these cytokines [39,47]. IL-2 is known to exert dual effects on T-cell activation depending on the immunological context [48,49]. This has been further supported by receptor-based studies on activated T-cells and regulatory T-cells [50]. Therefore, monitoring changes in IL-2 and TNF-α, in addition to IFN-γ, may be useful for the characterization of vaccine-associated immune responses in this model. IL-2 plays a central role in antigen-specific T-cell expansion and Th1 immune responses. Increased IL-2 production enhances macrophage-mediated bacterial killing, which contributes to the suppression of mycobacterial growth and is reflected by reduced CFUs.

This study was designed to establish a standardized, time-resolved framework for commonly used immune readouts following BCG vaccination rather than to define novel mechanisms or new correlates of protection. Accordingly, the present findings should be interpreted as descriptive reference data. In addition, the current analysis relied primarily on cytokine measurements and therefore does not fully capture the functional and cellular complexity of the immune response. Humoral immune responses, including antigen-specific IgG levels, were not evaluated in this study and may provide complementary information regarding vaccine-induced immunity. Specifically, flow cytometric analyses to identify cytokine-producing cell populations were not performed, and the activation of CD4+ and CD8+ T-cells, memory T-cell phenotypes, and regulatory T-cell responses were not evaluated despite the emphasis on IL-2. Additional studies incorporating detailed cellular immune profiling and humoral immune analyses will be required. Furthermore, in this study we utilized only the reference strain H37Rv, which, although widely used for standardization and comparability, does not fully represent the genetic and phenotypic diversity of clinical *M. tuberculosis* isolates, including MDR or hypervirulent strains. Immune response kinetics may differ when using recent clinical or drug-resistant isolates; therefore, future studies incorporating diverse strains will be necessary to strengthen the translational relevance of these findings.

## 5. Conclusions

Our findings suggest that immune responses following BCG vaccination are detectable from 4 weeks post-immunization, with temporal differences observed in cytokine expression profiles. Although a reduction in CFUs was observed across immunization periods, differences between the 4-, 6-, and 8-week groups were modest; therefore, the protective effect should be interpreted cautiously. While IL-2 levels increased at week 8, their biological significance in relation to protection remains unclear. Taken together, these results indicate that week 8 may represent a useful reference point for observing cytokine dynamics rather than a definitive optimal time for protection.

## Figures and Tables

**Table 1:** Glas-Col^®^ setting conditions.

No.	Stage	Time
1	Pre-heating	15 min
2	Nebulizer	30 min
3	Cloud decay	30 min
4	UV	15 min
5	Cool down	10 min
6	End	1 h 40 min

**Table 2:** Inflammation severity score.

Score	Inflammation Characteristic
0	Normal histological structure
1	Mild inflammatory cell infiltration in limited areas
2	Moderate inflammatory infiltration with focal exudate
3	Marked inflammatory infiltration in extensive areas
4	Severe inflammation with diffuse infiltration and structural destruction

## Data Availability

Data is contained within the article.

## Abbreviations

BCG	Bacillus Calmette–Guérin
LTBI	Latent tuberculosis infection
MDR-TB	Multidrug-resistant tuberculosis
ELISA	Enzyme-Linked Immunosorbent Assay
CFUs	Colony-Forming Units
H&E	Hematoxylin and Eosin Staining
IHC	Immunohistochemistry
PBS	Phosphate-Buffered Saline
OADC	Oleic acid–Albumin–Dextrose–Catalase
TB	tuberculosis
